# Magnetic properties of individual Co_2_FeGa Heusler nanoparticles studied at room temperature by a highly sensitive co-resonant cantilever sensor

**DOI:** 10.1038/s41598-017-08340-z

**Published:** 2017-08-21

**Authors:** Julia Körner, Christopher F. Reiche, Rasha Ghunaim, Robert Fuge, Silke Hampel, Bernd Büchner, Thomas Mühl

**Affiliations:** 1 Leibniz Institute for Solid State and Materials Research IFW Dresden, Helmholtzstr. 20, 01069 Dresden, Germany; 2 Institut für Festkörperphysik, Technische Universität Dresden, 01062 Dresden, Germany; 3 Center for Transport and Devices of Emergent Materials, Technische Universität Dresden, 01062 Dresden, Germany

## Abstract

The investigation of properties of nanoparticles is an important task to pave the way for progress and new applications in many fields of research like biotechnology, medicine and magnetic storage techniques. The study of nanoparticles with ever decreasing size is a challenge for commonly employed methods and techniques. It requires increasingly complex measurement setups, often low temperatures and a size reduction of the respective sensors to achieve the necessary sensitivity and resolution. Here, we present results on how magnetic properties of individual nanoparticles can be measured at room temperature and with a conventional scanning force microscopy setup combined with a co-resonant cantilever magnetometry approach. We investigate individual Co_2_FeGa Heusler nanoparticles with diameters of the order of 35 nm encapsulated in carbon nanotubes. We observed, for the first time, magnetic switching of these nanoparticles in an external magnetic field by simple laser deflection detection. Furthermore, we were able to deduce magnetic properties of these nanoparticles which are in good agreement with previous results obtained with large nanoparticle ensembles in other experiments. In order to do this, we expand the analytical description of the frequency shift signal in cantilever magnetometry to a more general formulation, taking unaligned sensor oscillation directions with respect to the magnetic field into account.

## Introduction

Magnetic nanoparticles are of high interest in many fields of research, reaching from magnetic storage techniques and spintronics^1, 2^ to biomedical applications which include drug delivery and hyperthermia treatment^3–5^. Recently, nanoparticles and -wires made of Heusler compounds have come into focus of research, due to their many potential applications in the aforementioned fields^6–8^. They can be grown with different aspect ratios, ranging from almost spherical nanoparicles to nanowires^1^, and might as well be encapsulated in carbon nanotubes to control size and shape of the particles^8^. First investigations have been carried out to analyze and understand the magnetic behavior of these Heusler nanomaterials^2, 6, 8–10^.

In order to successfully explore and employ the magnetic nanoparticles for the applications, a thorough characterization of their magnetic properties is essential. Several techniques are available for this purpose, for example SQUIDs^11–16^, magnetometers based on air coils driven by an alternating current or the Hall effect^17–19^, magnetic force microscopy-based methods^20, 21^, cantilever magnetometry^22–25^, spinpolarized scanning tunnelling microscopy^26, 27^, magnetometry based on nitrogen-vacancy defects^28^ and electron holography^1, 29^. These techniques have been employed to study magnetization reversal processes in nanoparticles induced by current^26, 27^ or by an external magnetic field^12, 21, 22^ and to investigate the magnetic stray fields of nanoparticles^1, 29^. Besides experimental techniques, theoretical simulations are used as well to gain insight into the nanoparticle’s magnetic behavior^30–34^.

The challenge for all experimental techniques lies in the nanometer size of the particles and the corresponding weak magnetic signals. Therefore, the measurement setups have to be very complex to reach the required sensitivity or spatial resolution, respectively^20^. This might be achieved by reducing the sensor’s dimensions^12, 22^, and by employing low temperatures^11, 21^.

In this publication we present a novel approach for experimentally studying magnetic nanoparticles based on cantilever magnetometry with two co-resonantly coupled cantilever beams. The strength of our approach is that it offers facile detection and very high sensitivity at the same time. Furthermore, the application of the sensor concept is not limited to cantilever magnetometry, but it may also be used for the detection of ultrasmall masses or magnetic field sensing.

In the following, we will briefly introduce the sensor concept, as well as the sensor fabrication. We then present magnetic measurements of a single carbon nanotube filled with a small number of individual Co_2_FeGa Heusler nanoparticles. We directly observed, to our knowledge for the first time, the magnetic switching of individual ferromagnetic Heusler nanoparticles at room temperature and with simple laser deflection-based cantilever magnetometry. This experiment opens the possibility of investigating the magnetic properties of nanoparticles with a fast and simple setup, providing high sensitivity and signal strength even at room temperature.

## Co-resonant concept in cantilever magnetometry

**Figure 1:**
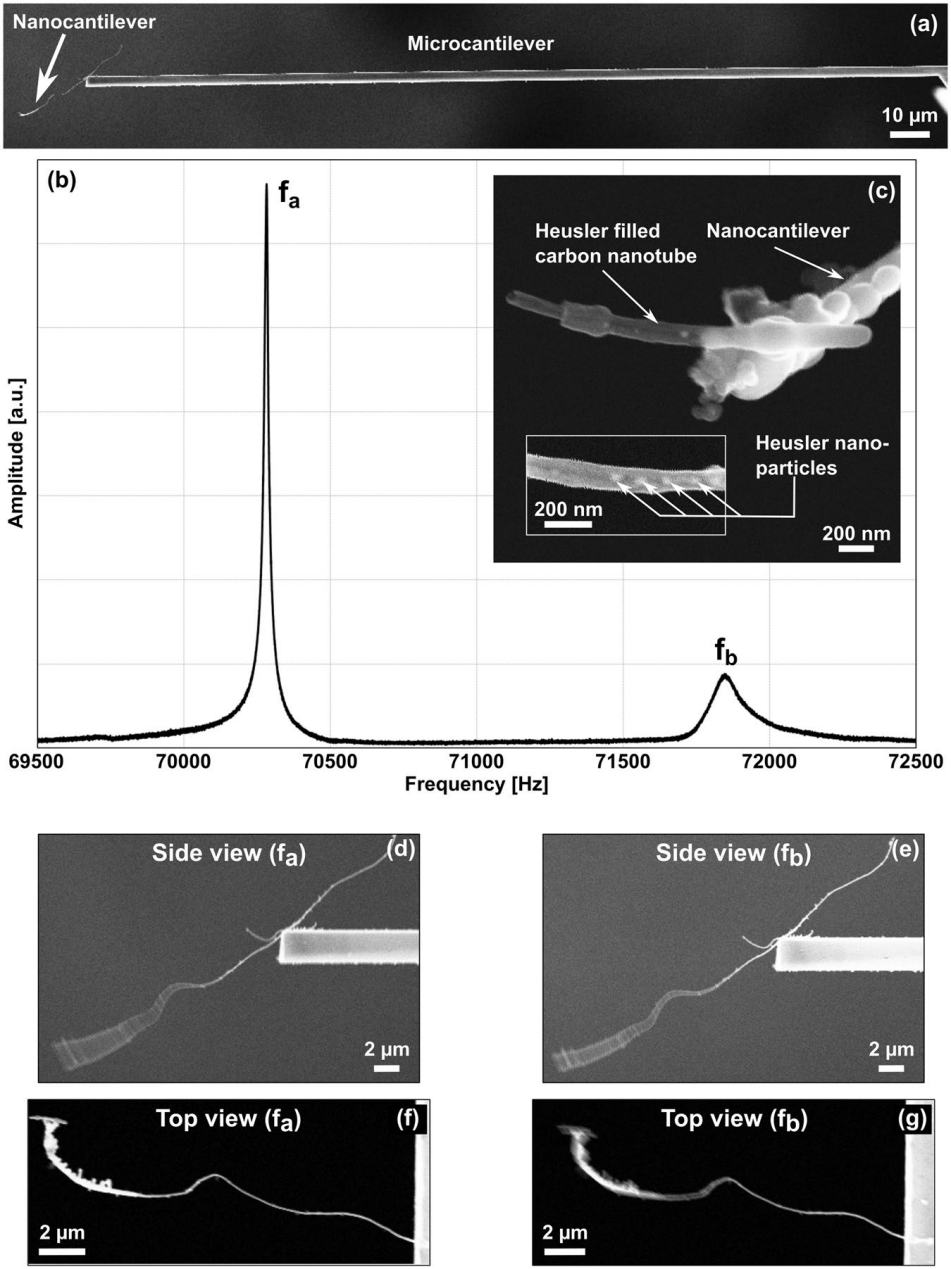
(**a**) Scanning electron microscopy (SEM) image of the co-resonantly coupled sensor consisting of a commercially available silicon microcantilever and a carbon nanotube as nanocantilever. (**b**) Amplitude response curve for the sensor obtained at the microcantilever which clearly shows the two resonance peaks with the frequencies *f*_*a*_ and *f*_*b*_. (**c**) Heusler filled carbon nanotube placed at the end of the nanocantilever. The inset shows a magnification where some of the Heusler particles are visible. (**d**) Side view of the oscillating nanocantilever at *f*_*a*_, (**e**) side view of the oscillating nanocantilever at *f*_*b*_, (**f**) top view of the oscillating nanocantilever at *f*_*a*_, (**g**) top view of the oscillating nanocantilever at *f*_*b*_ observed inside the SEM. The excitation parameters were the same for (**d**,**f**) as well as (**e**,**g**).

The concept of co-resonant cantilever magnetometry is based on the coupling of a micro- and a nanocantilever^35, 36^. The latter has cross sectional dimensions on the nanometer scale and a much shorter length than usual cantilevers. Downsizing of all spatial dimensions is associated with increased sensitivity. Various geometries are possible for the coupled system. However, for the experiment described here we used a sensor where the micro- and nanocantilever are coupled in succession as shown in Fig. 1a.

Besides the coupling, the eigenfrequencies of the two cantilevers are matched, leading to a co-resonantly coupled system, and therefore introducing a strong interplay between the cantilevers. Consequently, an interaction applied to the highly sensitive nanocantilever alters the oscillatory state of the coupled system as a whole and can be detected at the microcantilever with standard laser-optical methods. With the frequency matching, the sensitivity of the coupled system for external interactions like force and torque gradients is tremendously increased compared to the sensitivity of the single microcantilever. The underlying theoretical aspects of the behavior of the co-resonantly coupled system are discussed in depth in other publications^35, 37^ and therefore not presented here. Furthermore, first proof of principle experiments in cantilever magnetometry with iron filled carbon nanotubes as magnetic element^36, 38^ and scanning force microscopy^39^ demonstrated the vast potential of the co-resonant sensor concept in the study of magnetic phenomena.

Conventional cantilever magnetometry is a technique so study magnetic properties of samples by means of an oscillating one side clamped beam. The sample is placed at the free end of the cantilever and an external quasistatic magnetic field is applied. The magnetic interaction between this magnetic field and the sample exerts a torque on the cantilever, changing its oscillatory state. The cantilever’s oscillation can be detected by laser-optical methods, e.g. interferometry or deflectometry. The magnetostatic torque on the cantilever can be expressed as an additional spring constant Δ*k* in the system and therefore, the frequency shift Δ*f* of a cantilever modeled as a simple harmonic oscillator, is given by ref. 40:

1 $${\rm{\Delta }}f\approx {f}_{0}\frac{{\rm{\Delta }}k}{2{k}_{0}},$$

with the cantilever’s eigenfrequency *f*_0_ and spring constant *k*_0_. Thus, the interaction spring constant Δ*k* can be measured and evaluated to obtain magnetic information about the sample^23^.

For the co-resonantly coupled system, the situation is more complicated. First, we have two different cantilever beams with their own properties and hence spring constants, which are coupled. With the co-resonance and consequently the strong interplay between the two cantilevers, the amplitude response curve for the coupled system exhibits two resonance peaks corresponding to two modes of oscillation. These are each determined by a combination of the parameters of the single cantilevers, hence, effective sensor parameters for the two resonance modes need to be considered^37^. We will not discuss the details of the effective sensor parameters here and instead refer the reader to other publications^36, 37^ for an in-depth analysis. In the following it must only be kept in mind that a distinction between the properties of the single cantilevers and those of the coupled system is necessary and will be carried through in the following. We will denote the properties of the individual cantilevers by the indexes 1, 2 and those of the modes of the coupled system by *a*, *b*.

## Sensor fabrication and characteristics

We fabricate our sensors by employing electron beam and focused ion beam based milling and deposition techniques. A carbon nanotube (CNT) grown by aerosol-assisted chemical vapor deposition^41^ is used as nanocantilever and placed at the free end of a commercially available silicon microcantilever (Nanosensors TL-CONT, *NanoWorld AG*) by means of micromanipulation. The cantilevers are coupled in succession as depicted in a scanning electron image of the sensor in Fig. 1a. The CNT is fixed to the microcantilever by electron beam induced material deposition, creating a strong attachment point which can be described as a fixed clamping. Oscillation experiments have been carried out to determine the eigenfrequencies *f*_1,2_ of both individual cantilevers. Furthermore, together with geometric properties derived from SEM images, the dynamic spring constant of each individual cantilever *k*_1,2_ can be calculated according to ref. 42:

2 $${k}_{\mathrm{1,2}}\approx 1.03\cdot \frac{12{\pi }^{2}{f}_{1}^{2}{\rho }_{\mathrm{1,2}}{S}_{\mathrm{1,2}}{L}_{\mathrm{1,2}}}{{(1.875)}^{4}}.$$

Equation (2) is valid for the first flexural mode of the cantilevers which is used in our sensor. It only depends on the eigenfrequency *f*_1,2_, the mass density *ρ*_1,2_, cross sectional area *S*_1,2_ and length *L*_1,2_ of the micro- and nanocantilever, respectively. The relevant properties of both cantilevers are summarized in Table 1.

**Table 1:** Numerical values for the properties of micro- and nanocantilever before frequency matching.

Property	Microcantilever (1)	Nanocantilever (2)
Length *L*	(210 ± 6) *μ*m	(20.6 ± 0.6) *μ*m
Cross sectional area *S*	(6.17 ± 0.37) · 10^−11^ m^2^	(6.36 ± 0.94) · 10^−15^ m^2^
Eigenfrequency *f*	(70.4 ± 0.1) kHz	(348.6 ± 0.1) kHz
Spring constant *k*	(1.4 ± 0.3) N/m	(0.00034 ± 0.00009) N/m

The eigenfrequencies of the cantilevers are matched by electron beam induced material deposition at the free end of the nanocantilever, lowering its eigenfrequency to that of the microcantilever. The frequency matching results in a coupled system with two resonance frequencies, *f*_*a*_ = 70280.94 Hz and *f*_*b*_ = 71851.74 Hz, as depicted in the amplitude response curve in Fig. 1b. As stated above, the effective parameters of each resonance peak are a combination of the parameters of the individual cantilevers. This is especially important for the spring constants since these are necessary to evaluate measured frequency shift data, according to equation (1). The effective spring constants for each resonance peak are *k*_*a*_ = 0.002 N/m and *k*_*b*_ = 0.0004 N/m (see Methods section for derivation). Please note that these spring constants, especially *k*_*b*_, only slightly exceed the spring constant of the individual nanocantilever, illustrating the high sensitivity of the coupled system which is the consequence of the frequency matching.

Reducing the eigenfrequency of the nanocantilever from *f*_2_ = 348640 Hz to a value close to the eigenfrequency of the microcantilever *f*_1_ = 70435 Hz requires the deposition of a considerable amount of additional mass at the end of the nanocantilever compared to its own mass. We used platinum precursor gas to achieve the frequency matching so that the volume of the additional mass was kept small due to the high density of the platinum (see Fig. 1). However, the mass deposition can affect the oscillation direction of the nanocantilever^43^. Furthermore, we need to consider the effect of the mass deposition on the nanocantilever’s mode shape and, correspondingly, on its effective stiffness. For the first eigenmode however, this is not found to have a strong influence^44^. Even for our ratio of additional mass to total nanocantilever mass *m*_*add*_/*m*_2_ ≈ 5.5, the effective stiffness of the nanocantilever is only reduced by approximately 3%, which corresponds to the nanocantilver’s static spring constant. Hence, this can be considered by omitting the factor of 1.03 in equation (2).

In order to study the measured frequency shift data, we will have to take the nanocantilever’s oscillation direction as well as the geometry of the co-resonantly coupled sensor into account. Due to sensor fabrication the nanocantilever’s long axis and therefore its oscillation is misaligned to that of the microcantilever. Furthermore, a carbon nanotube exhibits an axial symmetry that is broken by structural features originating from the production process and the frequency matching by mass deposition^43^. This may result in a preferred oscillation direction of the carbon nanotube that is also not aligned to that of the microcantilever. Both effects reduce the effective coupling between the cantilevers and, consequently, result in a decrease of the measured frequency shift signal. To account for this deviation from the theory for an aligned system^35^, we derived the misalignment angles due to the geometry and preferred oscillation direction of the carbon nanotube from scannning electron microscopy (SEM) images. These images are depicted in Fig. 1d–g and clearly show different preferred oscillation directions for each resonance peak, hence the correction factors are calculated individually for both resonance peaks of the coupled system (see supplementary material). The correction factors found are ≈1.24 for resonance peak *f*_*a*_ and ≈0.94 for resonance peak *f*_*b*_, i.e. the measured frequency shift data would have to be multiplied with them. According to equation (2), the correction factors can also be combined with the effective spring constants for each resonance peak, leading to adjusted effective spring constants of ${k}_{a}^{\ast }=0.0027\,{\rm{N}}/{\rm{m}}$ and ${k}_{b}^{\ast }\approx {k}_{b}=0.0004\,{\rm{N}}/{\rm{m}}$. These are the characteristic effective spring constants for each resonance peak which are used for the data evaluation in the following.

Please note that the above mentioned broken symmetry of the carbon nanotube should in theory result in two fundamental flexural oscillation modes^43^. However, for the present sensor no experimental evidence of the second fundamental mode of the nanocantilever with a resonance frequency close to that of the first mode or to the microcantilever was found when studying the frequency matched sensor. Since a nanocantilever mode with a resonance frequency that is not matched to that of the microcantilever’s fundamental mode does not influence its sensing behavior^35^, it can be neglected. Therefore, the coupled harmonic oscillator model we employ for our calculations (see supplement) suffices. In case of an observable resonance frequency of the second fundamental mode of the nanocantilever close to the other resonance frequencies, the model would have to be extended to account for that.

The sensitivity limit for a co-resonantly coupled sensor with the above characteristics can be estimated by assuming the frequency shift induced by thermal noise as the ultimate limit. These considerations can be found in other publications^36^, and the resulting minimal detectable frequency shift for our sensor presented here is approximately 10^−7^ Hz. Although only an estimate, it demonstrates the possibility of studying even smaller magnetic nanoparticles with this method.

## Magnetometry setup

The magnetometry measurements were conducted in a *hr*-*MFM* (*NanoScan AG*) atomic force microscope at room temperature and under high vacuum. This equipment allows for an oscillation excitation of the sensor via a piezo shaker and the oscillation detection at the microcantilever by laser-deflection with a sectioned photo diode. The microscope features a phase-locked loop which allows for a precise measurement of the resonance frequency shift of the microcantilever. Furthermore, due to the geometric setup within the equipment, the cantilever is not positioned horizontally but tilted by an angle of 10°.

The external magnetic field was generated by means of an electromagnet. It is controlled through the input current which allows magnetic fields of up to ±650 mT. To measure the sensor’s response, the current has been swept by a programmed source to ensure uniform conditions for each measurement. However, to avoid thermal drifts throughout the measurement, the maximum peak value has only been applied for less than a second.

Two different kinds of measurements have been carried out: one where the magnetic field was swept through its whole range from zero to ±650 mT and back to zero (rate of 24 mT/s) and a second one where the magnetic field was only varied between zero and ±160 mT (rate of 2.7 mT/s). The latter prevents heating and therefore thermal drift of the setup and allows to resolve the frequency response in more detail.

To ensure that in case of the small magnetic field measurement all magnetic particles were in a defined magnetic state, the following procedure was carried out: application of +650 mT for less than one second, a field sweep from zero field to +160 mT and back to zero, another +650 mT pulse for less than one second and finally a field sweep from zero field to −160 mT and back to zero. The same steps were repeated for the field values with inverted signs to measure a complete magnetic hysteresis curve.

## Magnetic sample: Co_2_FeGa nanoparticles inside a carbon nanotube

The sample used in our experiment is an ensemble of carbon nanotubes filled with Co_2_FeGa Heusler nanoparticles. They have been fabricated according to the procedures described by Gellesch *et al*.^8^ followed by an additional annealing step (500 °C; 40 hours). This leads to a high yield of the desired phase of Co_2_FeGa nanoparticles inside the carbon nanotubes which has been confirmed by TEM-based nanobeam diffraction patterns and nuclear magnetic resonance measurements^8, 45^.

One of the nanotubes containing a small number of Heusler nanoparticles was transferred to the sensor’s nanocantilever, which is a carbon nanotube as well, by means of micromanipulation and fixed by electron beam-assisted material deposition (see methods section for details). This nanotube has a length of approximately (1.91 ± 0.06) *μ*m and contains six nanoparticles with diameters between approximately (25–60) nm (measured from SEM pictures). It would have been desirable to use a nanotube which only contains a single nanoparticle. However, in the many carbon nanotubes that were examined prior to the attachment procedure, no CNT with a single nanoparticle was observed and therefore one which only contained a small number of nanoparticles was chosen.

Figure 1c shows images of the filled nanotube attached to the end of the nanocantilever. After the sample had been affixed to the sensor, the resonance frequencies of the coupled system were remeasured to determine if the additional mass of the sample had altered them. They were found to be only slightly changed and therefore no further adjustment was necessary.

## Results

Before the magnetic sample was placed on the sensor, a control measurement with the empty sensor was carried out to ensure that it does not generate a magnetic signal itself. No frequency shift, except for a slight drift when the maximum magnetic field (650 mT) was applied for several seconds, was observed. The drift is easily explained by the heating of the measurement setup due to the high current trough the electromagnet. A nonmagnetic behaviour, i.e. no frequency shift related to the external magnetic field, is in agreement with the expected properties of the carbon nanotubes used as nanocantilever^41^.

The magnetometry measurements for the sensor with the sample attached were carried out consecutively for both resonance peaks of the coupled system by tracking the resonance frequency shift with the phase-locked loop. This is possible because each individual resonance peak exhibits a phase-response very similar to the one expected for a simple harmonic oscillator.

**Figure 2:**
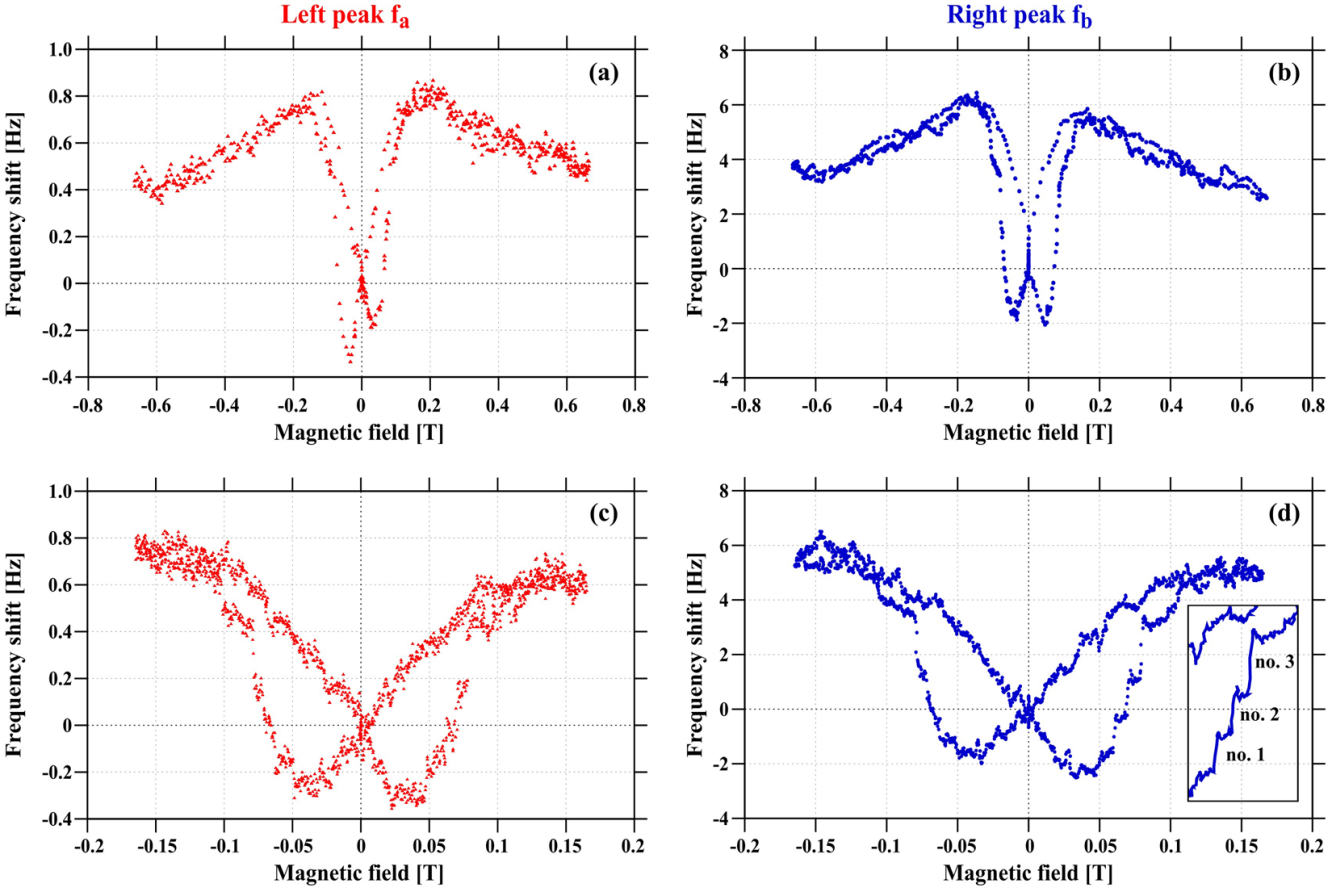
Field-dependent frequency shift signal for the co-resonantly coupled sensor obtained at the microcantilever by laser-deflection, (**a**) left-hand side resonance peak (*f*_*a*_) for the full range of external magnetic field, (**b**) right-hand side resonance peak (*f*_*b*_) for the full range of external magnetic field, (**c**) left-hand side resonance peak (*f*_*a*_) with higher resolution for external magnetic field of ±160 mT, (**d**) right-hand side resonance peak (*f*_*b*_) with higher resolution for external magnetic field of ±160 mT. The inset in (**d**) depicts part of the curve with the dots connected for better visualization of the jumps.

The magnetic field was swept to its maximum values of ±650 mT as well as to ±160 mT according to the measurement procedures described above. The frequency vs. magnetic field plots for both resonance peaks are depicted in Fig. 2.

Comparing the curves for both resonance peaks of the coupled system, we find that they show the same hysteretic behavior. However, the resonance peak with the smaller amplitude (peak b) shows a stronger frequency shift than the other one, hence it is more sensitive. This is an expected behavior of the co-resonantly coupled system^35^ and is also evident from the effective spring constants (${k}_{a}^{\ast }=0.0027\,{\rm{N}}/{\rm{m}}$, ${k}_{b}^{\ast }=0.0004\,{\rm{N}}/{\rm{m}}$) which differ by a factor of approximately seven. This agrees well with the relation between the magnitudes of the frequency shifts for the two resonance peaks.

The smaller peak b enables the observation of discrete jumps in the frequency shift which are evident in the higher resolved measurement with the smaller magnetic field range (Fig. 2d).

### Magnetic switching fields of individual Heusler nanoparticles

Such jumps indicate the magnetization reversal of individual single domain particles similar to cantilever magnetometry experiments for other nanosized materials^24^. The observed small jumps in the frequency shift depicted in Fig. 2d can be associated with magnetization reversal of the individual Heusler nanoparticles. This has been verified by repeating the measurements at different times and on several days and the jumps occurred reproducibly. If this was noise or thermal fluctuations these jumps would have appeared more randomly and also not only on the ascending branch of the curve.

We observe three clear jumps which should correspond to the biggest nanoparticles inside the nanotube depicted in Fig. 1c. One more jump might exist below and above the distinct ones but due to the signal noise it is not possible to state this with certainty. Therefore we will focus the following discussion on the three clear jumps. The magnetic field values where these jumps occur should directly correspond to the magnetic switching fields of the single nanoparticles, yielding 52 mT, 66 mT and 75 mT for jump number 1, 2 and 3, respectively. Please note that in general there is a strong dependence of the switching field on the angle between the magnetic field and anisotropy axis. The measured switching fields correspond to a particular angle which will be discussed below.

The values for the switching fields can be compared to another study where coercive fields of Co_2_FeGa nanoparticles encapsulated in carbon nanotubes have been investigated. In these experiments values of 22 mT (at room temperature) and 56 mT (at 5 K)^8^ have been found. The value for room temperature is significantly lower than the switching fields observed in our experiment. However, in the above mentioned publication, the nanoparticles were almost spherical, hence they are expected to have only a small coercive field. The nanoparticles we used in our experiment had been annealed for a very long time (40 hours), resulting in more ellipsoidal shaped particles. Details on the effect of annealing on the geometric properties of Co_2_FeGa Heusler nanoparticles are studied by Gellesch *et al*.^45^. Furthermore, in contrast to our experiment where we measured individual nanoparticles, the above cited experiments have been conducted for ensembles of nanotubes filled with a great number of Heusler nanoparticles with varying material properties.

Although the means of comparison for the obtained magnetic switching fields are limited, we conclude that we can derive reasonable values for our sample of Co_2_FeGa Heusler nanoparticles.

### Derivation of further magnetic properties

Besides the magnetic switching fields, further magnetic properties (magnetic moment *m*, anisotropy field *H*_*a*_) can be derived from the measured data. Finally, if the particles volumes are known, the magnetization and, if magnetocrystalline anisotropy is neglected, the particle’s aspect ratio can be determined.

By assuming cantilever magnetometry with a Stoner-Wohlfarth single domain magnetic particle with uniaxial anisotropy and the external magnetic field applied parallel to the easy axis, the frequency shift Δ*f* of the cantilever’s resonance frequency *f*_0_ can be related to the magnetic properties of the sample by refs 23, 24 and 36:

3 $$\frac{{\rm{\Delta }}f}{{f}_{0}}\approx \frac{{\mu }_{0}m}{2{k}_{0}{L}_{eff}^{2}}\cdot \frac{{H}_{ext}{H}_{a}}{{H}_{a}+{H}_{ext}}.$$

Here *m* = *M*_*s*_ *V* constitutes the magnetic moment of the sample (related to the saturation magnetization *M*_*s*_ and total sample volume *V*), *H*_*a*_ the anisotropy field, *H*_*ext*_ the externally applied magnetic field, *k*_0_ the cantilever’s spring constant and *L*_*eff*_ an effective cantilever length. The latter is dependent on the flexural mode and for the first bending mode it equals *L*_*eff*_ ≈ *L*/1.377^46^.

**Figure 3:**
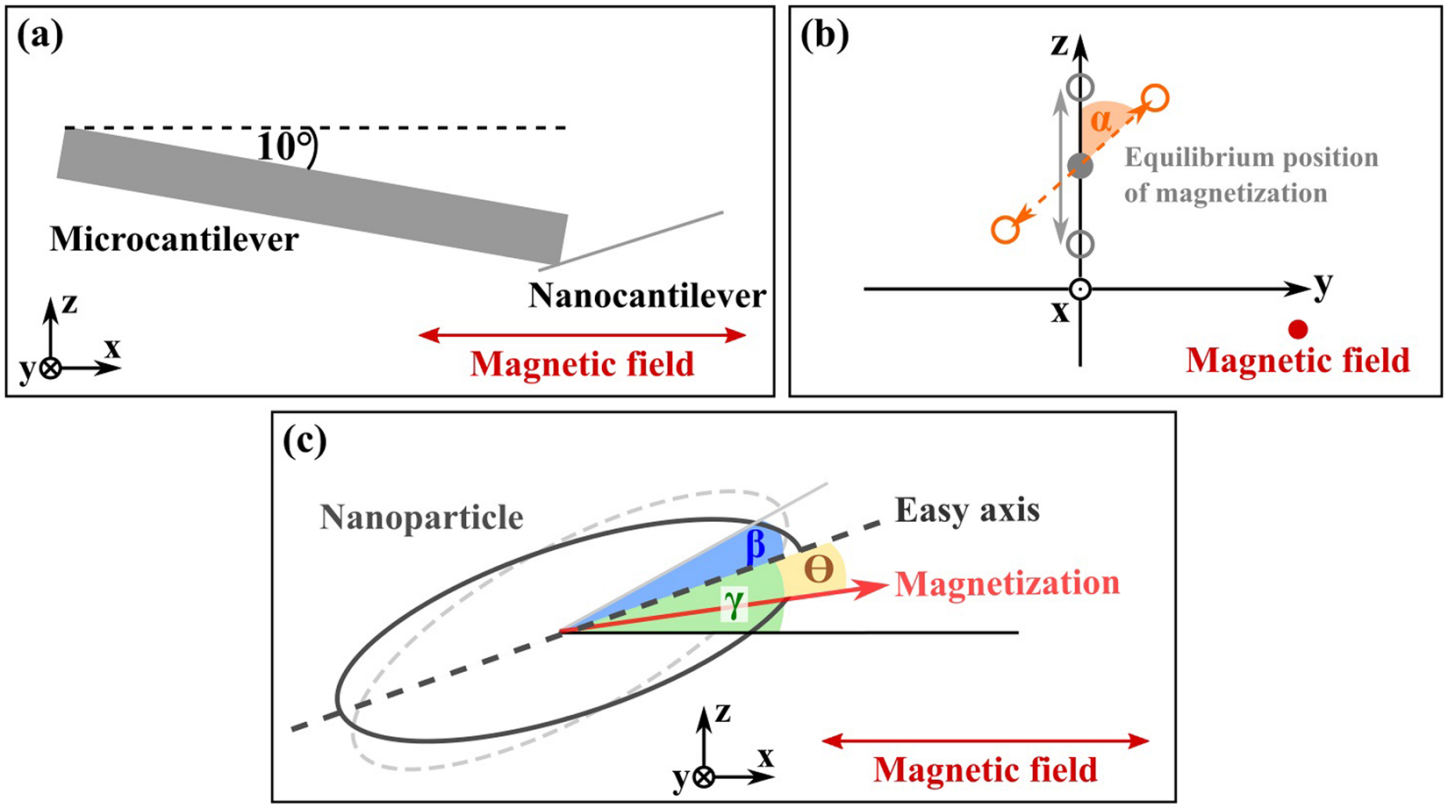
(**a**) Sketch of the sensor setup inside the equipment for the experiment in x-z-plane. (**b**) Magnetization in the y-z-plane. The grey circles indicate an oscillation in the x-z-plane and the orange circles depict the rotation of the oscillation plane by an angle *α* around the equilibrium position of the magnetization. (**c**) Definition of angles for the nanoparticle on the sensor exposed to a magnetic field. For clarity, the easy axis is defined as the equilibrium position which is tilted by the angle *γ* with respect to the external magnetic field. The angle *β* denotes the deflection angle due to sensor and sample oscillation and Θ is the canting of the magnetization away from the easy axis due to oscillation. Angles *β* and Θ are dependent on each other as discussed above. Please note that the axis of the nanocantilever in (**a**) and the particle easy axis in (**c**) do not necessarily coincide for an actual sample on a sensor.

However, equation (3) is not sufficient to describe the frequency shift in dependence on the external magnetic field for our experiment since the sample’s anisotropy axis is not aligned with the direction of the external field (see Fig. 3). Furthermore, as already discussed above, the oscillation plane of the nanocantilever is rotated with respect to the x-z-plane for resonance frequency *f*_*b*_ but not for *f*_*a*_ as top and side view SEM images of the oscillating sensor show (see Fig. 1d-g). We therefore need to include this misalignment and treat it accordingly, in this course deriving a more general expression for the frequency shift in cantilever magnetometry.

We start from the magnetic energy, assuming that Zeeman energy and shape anisotropy are dominant^6^. The Zeeman energy takes the angle between the sample’s magnetization and the external magnetic field into account. We define the external magnetic field to be oriented in the x-direction: $\overrightarrow{H}={H}_{ext}{\overrightarrow{e}}_{x}$. The equilibrium position of the sample’s easy axis is not aligned with the external magnetic field but tilted by an angle *γ* in the x-z-plane as depicted in Fig. 3c. Therefore, the magnetization $\overrightarrow{M}$ of the sample will already deviate from its easy axis by an angle Θ. If the sensor is oscillating in the x-z-plane with an oscillation angle *β*, the direction of the magnetization will change depending on *β*, leading to:

4 $$\overrightarrow{M}={M}_{s}\,\cos \,(\gamma +\beta -{\rm{\Theta }})\,{\overrightarrow{e}}_{x}+{M}_{s}\,\sin \,(\gamma +\beta -{\rm{\Theta }})\,{\overrightarrow{e}}_{z}.$$

To account for a rotation of the plane of oscillation as in the case of resonance peak *f*_*b*_, a rotation matrix $\hat{R}$ needs to be defined, with the normalized vector of the equilibrium position of the magnetization as the axis of rotation (*β* = 0 in equation (4)). The rotation angle of the plane of oscillation with respect to the x-z-plane is defined as *α* (see Fig. 3b). The rotation matrix has to be multiplied with the vector of magnetization and the vector for the external magnetic field, hence $\hat{R}\cdot \overrightarrow{M}\cdot \overrightarrow{H}$. The resulting Zeeman energy *E*_*z*_ is given by:

5 $$\begin{array}{rcl}{E}_{z} & = & -{\mu }_{0}{M}_{s}V{H}_{ext}\,[\cos \,\alpha \cdot \,\cos \,(\gamma +\beta -{\rm{\Theta }})+\tfrac{1}{2}\,(1-\,\cos \,\alpha )\,(\cos \,(\gamma -\beta -{\rm{\Theta }})\\  &  & +\,\cos \,(\gamma +\beta -{\rm{\Theta }}))].\end{array}$$

For the shape anisotropy energy, only the angle Θ between the magnetization and the easy axis is relevant and, hence:

6 $${E}_{a}=\frac{{\mu }_{0}}{2}V{M}_{s}^{2}\,({N}_{1}\,{\cos }^{2}\,{\rm{\Theta }}+{N}_{3}\,{\sin }^{2}\,{\rm{\Theta }})$$

with the demagnetization factors *N*_1_, *N*_3_.

The total magnetic energy considered is then:

7 $${E}_{mag}={E}_{z}+{E}_{a}.$$

The frequency shift relates to the magnetic energy by ref. 25:

8 $$\frac{{\rm{\Delta }}f}{{f}_{0}}={\frac{1}{2{k}_{0}{L}_{eff}^{2}}\cdot \frac{{\partial }^{2}{E}_{mag}}{\partial {\beta }^{2}}|}_{\beta =0}.$$

In order to insert equation (7) in equation (8), the angle Θ has to be expressed as a function of *β*. For an analytical solution this is only possible by using small angle approximations for Θ, hence the derived expression will only be valid for small external magnetic fields. The complete calculation can be found in the supplementary material as it offers no additional insight. Here we will only present the resulting expression for the frequency shift which reads:

9 $$\begin{array}{rcl}\frac{{\rm{\Delta }}{f}_{a,b}}{{f}_{a,b}} & = & K\cdot [(1-2\,\cos \,\alpha {C}_{2}+{C}_{2}^{2})\cdot {H}_{ext}\cdot \,\cos \,(\gamma -{C}_{1})\\  &  & +{C}_{2}^{2}\cdot {M}_{s}\cdot ({N}_{3}-{N}_{1})\cdot \,\cos \,(2{C}_{1})]\end{array}$$

with

10 $$K=\frac{{\mu }_{0}V{M}_{s}}{2{k}_{a,b}^{\ast }{L}_{eff}^{2}}$$

11 $${C}_{1}=\frac{{H}_{ext}\cdot \,\sin \,\gamma }{{H}_{ext}\cdot \,\cos \,\gamma +{M}_{s}\,({N}_{3}-{N}_{1})}$$

12 $${C}_{2}=\,\cos \,\alpha \cdot \frac{{H}_{ext}^{2}+{M}_{s}\,({N}_{3}-{N}_{1})\cdot {H}_{ext}\cdot \,\cos \,\gamma }{{({H}_{ext}\cdot \cos \gamma +{M}_{s}({N}_{3}-{N}_{1}))}^{2}}.$$

Please note that the resonance frequencies *f*_*a*,*b*_ as well as the effective spring constants ${k}_{a,b}^{\ast }$ for each resonance peak of the coupled system are used. Furthermore, the expression *M*_*s*_(*N*_3_ − *N*_1_) can be substituted by the anisotropy field *H*_*a*_ and *VM*_*s*_ by the magnetic moment *m*. Doing so, equation (3) can be derived from expression (9) if *γ* and *α* equal zero.

We applied equation (9) with the above mentioned substitutions to our measured data and fitted the magnetic moment *m*, anisotropy field *H*_*a*_ and the angle *γ*. The rotation angle *α* of the oscillation plane was obtained from SEM images by evaluating the top and side view of the oscillating sensor for both resonance peaks (see Fig. 1d–g). For resonance peak *f*_*a*_ a rotation angle of *α*_*a*_ = 0° and for peak *f*_*b*_ *an angle of α*_*b*_ = 35° was found and employed for the fits.

Please note that the angle obtained from the SEM images would actually be the angle of oscillation plane rotation around the easy axis of the sample and not the rotation around the equilibrium position of the magnetization. However, for our geometry this difference is small and below the uncertainty of the angle determination. We therefore use this angle from SEM images as the plane rotation angle *α*.

**Figure 4:**
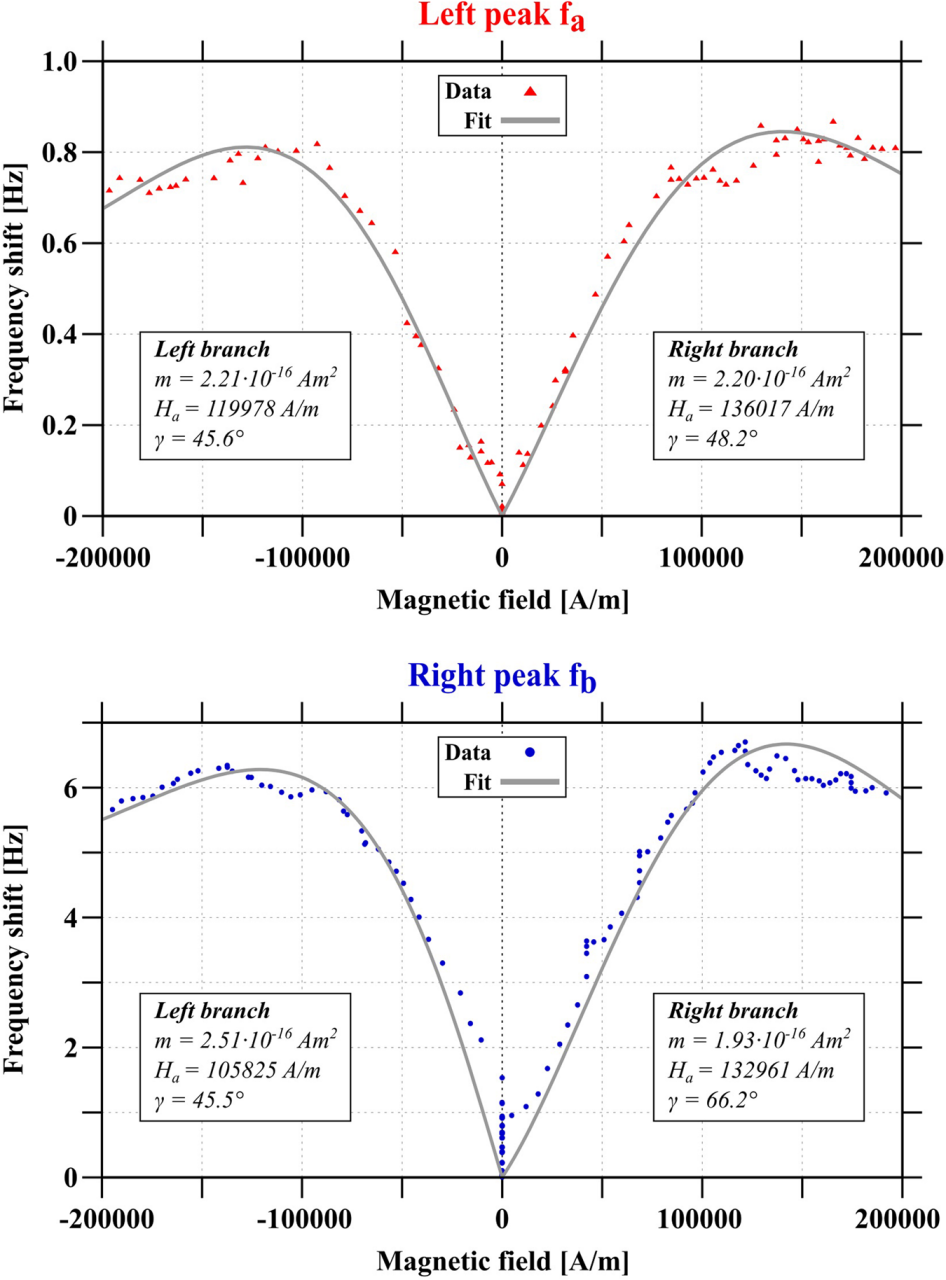
Exemplary frequency shift data for the two resonance peaks of the coupled system fitted with equation (9). Please note that the fit is only valid for small external magnetic fields and was furthermore done for each branch separately. The corresponding parameters are given and show a good agreement. For this figure the unit A/m was chosen to correspond to the theoretical derivation. Experimental data in Fig. 2 is given in T.

The fit was carried out for both resonance peaks for the sweep direction without indication of jumps in the frequency shift signal and only for small external magnetic fields, i.e. *H*_*ext*_ < 200 kA/m. Exemplary fits are shown in Fig. 4 and the resulting fit values are summarized in Table 2. The results are mean values from fitting the frequency shift data of six consecutive measurements for each resonance peak and a good consistency is found between the fit values for the magnetic properties for both resonance peaks. The larger deviation for the angle *γ* can be explained by the high uncertainty of the determination of the rotation angle *α* from SEM images.

**Table 2:** Magnetic and geometric properties of the nanoparticles, obtained by fitting the frequency shift signal for both resonance peaks *f*_*a*_ and *f*_*b*_ and both field directions with equation (9). The fit values are averaged for fits of six consecutive measurements for each peak.

Parameter	Left peak *f*_*a*_	Right peak *fb*
Magnetic moment *m*	(2.3 ± 0.1) · 10^−16^ Am^2^	(2.4 ± 0.3) · 10^−16^ Am^2^
Anisotropy field *μ*_0_ *H*_*a*_	(151 ± 12) mT	(147 ± 15) mT
Geometric angle *γ*	(46 ± 2) deg	(54 ± 11) deg
Demag. factor *N*_1_	0.280 ± 0.006	0.287 ± 0.005
Demag. factor *N*_3_	0.360 ± 0.003	0.357 ± 0.003
Aspect ratio Λ	1.29	1.24

.

The obtained value for the magnetic moment *m* ≈ 2.3 · 10^−16^ Am^2^ can be further analyzed in order to get an estimate for the saturation magnetization *M*_*s*_. However, this requires some assumptions regarding the geometry of the nanoparticles. The magnetization and magnetic moment are related through the volume of the magnetic sample, so in our case the total volume of *N* = 6 nanoparticles enclosed in the carbon nanotube needs to be considered. The number of nanoparticles is determined from SEM pictures. However, these images only allow for a very rough measurement of the diameter of the nanoparticles. Since they are furthermore not spherical but exhibit a slightly elongated shape^45^, we will use an average particle diameter of *d*_*avg*_ = (40 ± 7) nm based on values obtained from Gellesch *et al*. through TEM studies of ensembles of these Co_2_FeGa filled nanotubes^8, 45^. With *m* = *VM*_*s*_ and *V* = *N* · 4/3*π*(*d*_*avg*_/2)^3^ we can determine a magnetization of *μ*_0_ · *M*_*s*_ = (1.5 ± 1.0) T. This value has a very high uncertainty, mainly due to the wide variance of the particle diameter. However, the value for the magnetization is in the expected range as the few other studies for these nanoparticles indicate^8^. We therefore conclude that we could derive a sensible value for the magnetization.

Besides the magnetic properties, geometric information about the nanoparticles and the sensor are also derived from the fit. As stated above, the anisotropy field is related to the magnetization and the demagnetization factors by *H*_*a*_ = *M*_*s*_(*N*_3_ − *N*_1_). By assuming particle shapes that correspond to prolate ellipsoids, hence *N*_2_ = *N*_3_, and applying *N*_1_ + *N*_2_ + *N*_3_ = 1^47^, the aspect ratio Λ of the particles can be calculated, yielding Λ ≈ 1.27 ± 0.03^48^. This agrees with the findings from Gellesch *et al*. who studied the effect of the annealing time on the particle aspect ratio and found *λ* ≈ 1.1 for an annealing time of 40 hours^45^.

Another fit parameter is the angle *γ* which shows a rather large deviation for the two resonance peaks. As stated above this originates from the high uncertainty in determining the angle of the plane rotation for resonance peak *f*_*b*_. Hence, the fit value of *γ* ≈ 46° for resonance peak *f*_*a*_ where no rotation of the oscillation plane occured, gives a more accurate value.

Finally, it is instructive to compare our co-resonant magnetometry to conventional cantilever magnetometry employing a single standard cantilever. If our sample would simply be attached to the microcantilever that forms one component of our setup (*k*_1_ = 1.4 N/m, see Table 1), the magnitude of the Δ*f*_*a*_ signal (0.8 Hz range, see Fig. 2) would be reduced by a factor of approximately 50,000. This estimation is based on equation (8) where the second derivative of the magnetic energy would be unchanged but regarding *k*_0_ and *L*_*eff*_ the corresponding values of the microcantilever would be used. If we compare with Δ*f*_*b*_ the signal reduction would be even larger. Of course, the signal reduction would be less dramatic for conventional cantilever magnetometry if much softer cantilevers with a spring constant in the *μ*N/m range would be used^22^. However, these cantilevers require a sophisticated design and have been optimized for many years. In contrast to that, our co-resonant method was only recently introduced and has a lot of potential for improvement, especially by tailoring the nanocantilever to offer a much higher sensitivity compared to the sensor we present here.

## Conclusion

The above considerations show that in order to obtain reliable magnetic information from these measurements, it is very important to take all aspects of the sensor and the oscillation geometry into account. We demonstrated this by expanding the well-known expression for the frequency shift in cantilever magnetometry to a more general description of the problem. With deceasing sensor size and hence, an increased susceptibility of the sensor to smallest influences which can alter its structure and its oscillation properties, these considerations will become valuable.

In our experiment we demonstrated that we could derive sensible values for the magnetic and geometric properties of individual Co_2_FeGa nanoparticles. It shows the way how to obtain magnetic information of individual nanoparticles from data measured by co-resonant cantilever magnetometry. However, this is only a first approach which contains reasonable assumptions and simplifications where the derivation of equation (9) is concerned. For future experiments, the evaluation methods can be refined to obtain even more accurate data, e.g. by employing numerical solutions, and also an optimization of the sensor geometry can be considered. A combination of cantilever magnetometry with TEM investigation of the nanoparticles would also increase the accuracy of data evaluation.

The point we want to stress here is that we have applied a simple room temperature cantilever magnetometry setup based on laser-deflection oscillation detection of a microcantilever to measure magnetic properties of Co_2_FeGa Heusler nanoparticles encapsulated in a carbon nanotube. The uniqueness of this approach lies in its simplicity, since no special conditions (e.g. low temperatures, clean surfaces), except high vacuum, are required. The approach is suitable for all kinds of magnetic nanoparticles which can either be transferred to the sensor or even grown on it, therefore spanning a broad range of possible applications. Furthermore, it is not limited to magnetometry but can be used in various cantilever-based techniques.

## Methods

### Micromanipulation

The sensor fabrication and sample attachment is done with a micromanipulator (*Kleindiek Nanotechnik GmbH*) equipped with a tungsten needle. With this, the empty nanotube, i.e. the nanocantilever, as well as the Heusler filled CNT are picked up, slightly fixed to the needle by electron beam induced carbon deposition and transferred to the desired location. There, the nanotube/sample is attached again, this time more strongly by electron beam induced material deposition. By removing the tungsten needle, the inital bond between needle and nanotube/sample is broken and it is fixed at the desired position.

### Vibration experiments

The sensor characterization is carried out with a custom-made vibration stage inside the scanning electron microscope. It is equipped with a holder for the sensor and a piezo plate along with a connector to supply an AC voltage to the piezo. This setup allows direct observation of the micro- and nanocantilever oscillation and *in*-*situ* frequency matching. Furthermore, resonance curves are obtained from SEM pictures and with a Lorentzian fit applied to the quadratic amplitude values one can obtain the resonance frequency for the oscillation. With these properties and the geometry of the sensor, the spring constants are calculated according to equation (2).

### Determination of effective spring constants for the coupled system

In order to determine the effective spring constants for the two resonance peaks of the coupled system, the system is simulated as a coupled harmonic oscillator model with the measured and known properties of the single systems. Simulation with and without an arbitrary but sufficiently small interaction spring constant *k*_3_ gives a frequency shift Δ*f*_*a*,*b*_ for each resonance peak. Employing and rearranging equation (1) for each resonance peak allows for the determination of *k*_*a*,*b*_ which are the effective spring constants for both resonance peaks. In order to get a reliable solution, the interaction spring constant *k*_3_ used for the calculation has to be at least two orders of magnitude smaller than the smallest single spring constant in the system. Further information on effective spring constants can be found elsewhere^36, 37^.

### Nanotube preparation

The nanotubes used as nanocantilever have been fabricated by aerosol-assisted chemical vapor deposition. A solution of ferrocene and cyclohexan has been used as precursor and the droplets obtained with ultrasonication have been transported to the substrate by argon gas flow. The tubes are growing perpendicular to the substrate at a temperature around 800 °C and are reaching lengths of up to 1 mm. Afterwards, the nanotubes are removed from the subtrate and tempered at 2500 °C for an hour to ensure that all katalytic iron particles are evaporated, leaving dimagnetic nanotubes. Next, the CNTs are dispersed by ultrasonication leading to breaking of the tubes which results in lengths below 40 *μ*m. These tubes are then dispersed on a TEM grid from where single nanotubes can be picked up.

### Sample preparation and transfer

After fabrication and characterization, the nanotubes containing the Heusler nanoparticles have been dispersed on a lacey carbon grid. From there, a single nanotube has been chosen and transferred to the sensor by means of micromanipulation. The nanotube has been chosen according to the following criteria: (i) length between (1–2) *μ*m to allow for the manipulation (minimum length) while at the same time limiting its mass (maximum length) and therefore the influence on the nanocantilever; (ii) clean outer surface, i.e. no particles or contamination on the outside of the nanotube.

### Data availability

The datasets generated and/or analysed during the current study are available from the corresponding author on reasonable request.

## Electronic supplementary material

Supplementary Material
