# Supplementary material for: Magnetic properties of individual Co2FeGa Heusler nanoparticles studied at room temperature by a highly sensitive co-resonant cantilever sensor
Source: Sci Rep. 2017 Aug 21;7:8881. doi: 10.1038/s41598-017-08340-z (PMC5566407; doi:10.1038/s41598-017-08340-z)
Supplement: Supplementary file 1 — Supplementary Material [file 41598_2017_8340_MOESM1_ESM.pdf]

Supplementary Material

Magnetic properties of individual Co_2FeGa Heusler nanoparticles studied at room temperature by a highly sensitive co-resonant cantilever sensor

Julia Körner[1], Christopher F. Reiche[1], Rasha Ghunaim[1], Robert Fuge[1], Silke Hampel[1], Bernd Büchner[1,2] and Thomas Mühl[1]

[1] Leibniz Institute for Solid State and Materials Research IFW Dresden, Helmholtzstr. 20, 01069 Dresden, Germany
[2] Institut für Festkörperphysik, Technische Universität Dresden, 01062 Dresden, Germany

Corresponding author:
Dr. Thomas Mühl
E-Mail: t.muehl@ifw-dresden.de

A: Correction for non-aligned co-resonant oscillations

The theoretical description of the co-resonant sensor concept used in the present and previous work [1, 2, 3] is based on a one-dimensional coupled harmonic oscillator model. This simple model can only be applied, however, if the oscillation directions of the two beam-like structures of a co-resonant sensor are aligned to each other. In a real sensor, like the one used in the main publication, this is usually not the case.
In order to still derive quantitative data with the simple harmonic oscillator model, a modification is necessary [4]. Essentially, the misalignment of the oscillation directions of the beam-like structures can be treated by using an angled coupled harmonic oscillator model (see figure 1 (a)). In this model the two masses (m_1,m_2) are still subject to one-dimensional movements individually that are, however, not aligned along the same direction.
Consequently, the projection of a m_1 displacement on the m_2 oscillation direction is smaller than the m_1 displacement itself. The same applies to the projection of the m_2 displacement on the m_1 oscillation direction. Other than the projection of the m_1 displacement, the force generated by k_2 acts fully on m_2. However, in addition to the projection of the m_2 displacement, only a projection of the k_2-induced force acts on m_1. All reductions by these projections can be described by the factor ϵ. Thus, misalignment of the two subsystems' oscillation directions does not change their individual resonance frequencies but leads to a reduction of the coupling of the two oscillators.
If damping is neglected, the equations of motion for the thus modified problem can be written in matrix form:

$$\mathbf{M} \cdot \ddot{\vec{s}}(t) + \mathbf{K} \cdot \vec{s}(t) = \vec{F}(t) \tag{1}$$

with the matrices

$$\mathbf{M} = \begin{pmatrix} m_1 & 0 \\ 0 & m_2 \end{pmatrix}, \quad \mathbf{K} = \begin{pmatrix} k_1 + \epsilon^2 k_2 & -\epsilon k_2 \\ -\epsilon k_2 & k_2 + k_3 \end{pmatrix}, \tag{2}$$

the deflection of the masses from their equilibrium position $\vec{s} = (s_1, s_2)$ and the excitation force vector $\vec{F}(t) = (F_0, 0)\cos(\omega_D t)$. Here, m_i and k_i are the effective masses and spring constants for the two partial mechanical resonators and k_3 describes an external force gradient acting on the smaller subsystem.

From these equations it is possible to calculate the amplitudes and phases of the partial systems as homogenous solutions by using a complex ansatz $\vec{s}(t) = (A_1, A_2)\exp(i\omega t)$. In this work, however, only the resonance frequencies of the coupled system are of interest. They can be derived by using this ansatz in equation (1), which results in a system of equations for the complex amplitudes. The resonance frequencies can be derived by demanding a non-trivial solution, therefore requiring the determinant of the coefficient matrix to vanish. This results in the resonance frequencies of the coupled system:

$$\omega^2(\epsilon, k_3)_{a/b} = \frac{T}{2} \mp \sqrt{\left(\frac{T}{2}\right)^2 - \frac{\omega_1^2 \omega_2^2}{k_1}\left(k_1 + \frac{k_1 k_3}{k_2} + \epsilon^2 k_3\right)} \tag{3}$$

with

$$T := \omega_1^2 \left(1 + \epsilon^2 \frac{k_2}{k_1}\right) + \omega_2^2 \left(1 + \frac{k_3}{k_2}\right). \tag{4}$$

To derive these equations, $m_i = k_i/\omega_i^2,\ i = (1, 2)$ was employed.

With these results it can be understood that each misalignment of the oscillation directions leads to a change of the frequency shift of the respective mode $\Delta\omega_{a/b}$ which corresponds to adjusted effective spring constants $k^*_{a,b}$.

By introducing a correction factor

$$\eta(\epsilon, k_3) = \frac{k^*_{a,b}}{k_{a,b}} = \frac{\Delta\omega_{a/b}(0, k_3)}{\Delta\omega_{a/b}(\epsilon, k_3)} = \frac{\omega_{a/b}(0, k_3) - \omega_{a/b}(0, 0)}{\omega_{a/b}(\epsilon, k_3) - \omega_{a/b}(\epsilon, 0)} \tag{5}$$

this effect can be included into effective spring constants that result from calculations based on an aligned harmonic oscillator model ($k_{a,b}$). Please note that for sufficiently small k_3 this correction factor depends only on ϵ:

$$\eta(\epsilon, k_3) \approx \eta(\epsilon), \quad k_3 << k_2 \tag{6}$$

For the co-resonant sensor used for the experiments in this work the geometric reduction factor can be described by $\epsilon = \cos(\varphi)\cos(\xi)$. The factor $\cos(\varphi)$ originates from the structural inclination of the long axis of the nanocantilever with respect to that of the microcantilever (see figure 1(b)) as can be derived from scanning electron microscopy (SEM) images taken from the side view perspective. This results in an angle of $\varphi \approx 31°$, which does not change during the experiment.
The contribution $\cos(\xi)$ describes the non-aligned preferred oscillation direction of the nanocantilever with respect to that of the microcantilever due to its disturbed axial symmetry (see figure 1(c)). In order to derive the angle ξ, the sensor was excited to resonant oscillations and observed from side and top view inside the SEM with identical excitation parameters. From the SEM images the projection of the resonant oscillation amplitude of the nanotube in the microscope's imaging plane was measured for both views. The angle ξ was then calculated from these amplitude projections by simple trigonometry. Please note that this angle is different for both resonant modes of the coupled system and was measured independently for each respective mode. This resulted in $\xi_a \approx 0°$ and $\xi_b \approx 35°$.
Finally, the condition of a sufficiently small k_3 is well satisfied in these experiments, so the correction factors from equation (5) for both combined resonance modes ($\eta(\epsilon_a) \approx 1.24$, $\eta(\epsilon_b) \approx 0.94$) are constant and were used to calculate the adjusted effective spring constants $k^*_{a/b}$.

B: Derivation of cantilever frequency shift from magnetic energy

The following derivation is mostly based on [5, 6, 7].

We define the external magnetic field to be oriented along the x-axis, i.e. $\vec{H} = H_{ext}\vec{e_x}$, and the sensor equilibrium position to be in the x-z-plane. The sensor oscillation plane is tilted by an angle α with respect to the x-z-plane (see figure 2 in the main paper for angle definitions). Furthermore, we assume the magnetic energy of the system to be dominated by Zeeman and shape anisotropy energy.
The Zeeman energy is given by:

$$E_z = -\mu_0 \int \vec{M} \cdot \vec{H} dV \quad . \tag{7}$$

If the easy axis of the magnetic sample is not aligned with the external magnetic field but tilted by an angle γ, the magnetization $\vec{M}$ already cants away from the easy axis by an angle Θ at the equilibrium position. With a sensor oscillation of an angle β in the x-z-plane, the magnetization vector is:

$$\vec{M} = M_s \cos(\gamma + \beta - \Theta) \vec{e_x} + M_s \sin(\gamma + \beta - \Theta) \vec{e_z} \quad . \tag{8}$$

To take a rotation of the oscillation plane into account, $\vec{M}$ is multiplied with a rotation matrix $\hat{R}$ which is defined by the rotation angle α and an axis of rotation. The latter is given by the unit vector of the equilibrium position of the magnetization: $\vec{M}/M_s = \cos\gamma'\vec{e_x} + \sin\gamma'\vec{e_z}$, with $\gamma' = \gamma - \Theta$. Hence:

$$\hat{R} = \begin{pmatrix} \cos\alpha + \cos^2\gamma' \cdot (1 - \cos\alpha) & -\sin\gamma' \sin\alpha & \cos\gamma' \sin\gamma' \cdot (1 - \cos\alpha) \\ \sin\gamma' \sin\alpha & \cos\alpha & -\cos\gamma' \sin\alpha \\ \cos\gamma' \sin\gamma' \cdot (1 - \cos\alpha) & \cos\gamma' \sin\alpha & \cos\alpha + \sin^2\gamma' \cdot (1 - \cos\alpha) \end{pmatrix} \tag{9}$$

Application of $\hat{R}$ on $\vec{M}$ (no y-component), scalar multiplication with $\vec{H}$ (only x-component) and employing the result into equation (7) yields the Zeeman energy:

$$E_z = -\mu_0 M_s V H_{ext} \left[\cos\alpha \cdot \cos(\gamma + \beta - \Theta) + \frac{1 - \cos\alpha}{2} \left\{ \cos(\gamma - \beta - \Theta) + \cos(\gamma + \beta - \Theta) \right\} \right] \tag{10}$$

with the saturation magnetization M_s and the sample volume V.

For the shape anisotropy energy of prolate spheroids, only the angle between the easy axis and the magnetization is relevant, hence:

$$E_a = \frac{\mu_0}{2} V M_s^2 \left(N_1 \cos^2\Theta + N_3 \sin^2\Theta \right) \tag{11}$$

with the demagnetization factors N_1, N_3 and, therefore, the total magnetic energy is:

$$E_{mag} = E_z + E_a \quad . \tag{12}$$

First, the angle Θ needs to be expressed as a function of β. In order to achieve this, the derivative of the magnetic energy with respect to Θ is set to zero:

$$\frac{\partial E_{mag}}{\partial \Theta} = 0 = -H_{ext}\left(\cos\alpha\sin\left(\gamma+\beta-\Theta\right) + \frac{1-\cos\alpha}{2}\left\{\sin(\gamma-\beta-\Theta)+\sin(\gamma+\beta-\Theta)\right\}\right)$$
$$+ M_s\left(N_3-N_1\right)\sin\Theta\cdot\cos\Theta \quad . \tag{13}$$

Furthermore, the second derivative of the magnetic energy with respect to Θ, $\partial^2 E_{mag}/\partial\Theta^2$, needs to be positive.

Applying addition theorems for $\sin(\gamma \pm \beta - \Theta)$, $\sin(\pm\beta - \Theta)$ and $\cos(\pm\beta - \Theta)$ to isolate γ and furthermore using a small angle approximation for the oscillation angle β which yields $\sin\beta \approx \beta$ and $\cos\beta \approx 1$, leads to an expression for $\beta(\Theta)$:

$$\beta(\Theta) = \frac{M_s(N_3-N_1)\sin\Theta + H_{ext}\left(\cos\gamma\cdot\tan\Theta - \sin\gamma\right)}{H_{ext}\cos\alpha\left(\cos\gamma + \sin\gamma\cdot\tan\Theta\right)} \quad . \tag{14}$$

Finding an expression for Θ by using equation (14) is only possible, if a small angle approximation is applied for Θ. Since Θ denotes the deviation of the magnetization direction from the easy axis, this is only valid for small external magnetic fields. In that case, $\sin\Theta \approx \Theta$ and $\tan\Theta \approx \Theta$ are used. If a more accurate solution is desired, a numerical solution is necessary at this point.

With the above approximations, equation (14) becomes:

$$\beta(\Theta) \approx \frac{\Theta\left(M_s(N_3-N_1) + H_{ext}\cos\gamma\right) - H_{ext}\sin\gamma}{H_{ext}\cos\alpha\left(\Theta\sin\gamma + \cos\gamma\right)} \quad . \tag{15}$$

Now, Θ can be expressed as a function of β by using a Taylor series expansion:

$$\Theta = \Theta\bigg|_{\beta=0} + \frac{\partial\Theta}{\partial\beta}\bigg|_{\beta=0}\cdot\beta = \Theta_0 + \left(\frac{\partial\beta}{\partial\Theta}\bigg|_{\Theta_0}\right)^{-1}\cdot\beta. \tag{16}$$

Evaluation of equations (15) and (16) finally yield:

$$\Theta(\beta) = \frac{H_{ext}\sin\gamma}{M_s(N_3-N_1) + H_{ext}\cos\gamma} + \cos\alpha\cdot\frac{H_{ext}^2 + M_s(N_3-N_1)H_{ext}\cos\gamma}{\left(M_s(N_3-N_1) + H_{ext}\cos\gamma\right)^2}\cdot\beta. \tag{17}$$

Equation (17) can be inserted into expression (12) for the magnetic energy. Calculating the second derivative of the magnetic energy with respect to β leads to the frequency shift for cantilever magnetometry as presented in the main paper.

References

[1] C. F. Reiche, J. Körner, B. Büchner, and T. Mühl. Bidirectional scanning force microscopy probes with co-resonant sensitivity enhancement. In *Proceedings of the 15th IEEE International Conference on Nanotechnology, Rome*, page 1222, 2015.

[2] J. Körner, C. F. Reiche, B. Büchner, G. Gerlach, and T. Mühl. Application of a co-resonant sensor concept in cantilever magnetometry. In *Proceedings of the 15th IEEE International Conference on Nanotechnology, Rome*, page 1239, 2015.

[3] C. F. Reiche, J. Körner, B. Büchner, and T. Mühl. Introduction of a co-resonant detection concept for mechanical oscillation-based sensors. *Nanotechnology*, 26(33):335501, 2015.

[4] C. F. Reiche. *Novel Sensors for Scanning Force Microscopy Based on Carbon Nanotube Mechanical Resonators*. PhD thesis, Technische Universität Dresden, Fakultät Mathematik und Naturwissenschaften, 2015.

[5] D. P. Weber. *Dynamic Cantilever Magnetometry of Individual Ferromagnetic Nanotubes*. PhD thesis, Universität Basel, Fakultät für Naturwissenschaften, 2014.

[6] U. Gysin, S. Rast, A. Aste, T. Speliotis, C. Werle, and E. Meyer. Magnetic properties of nanomagnetic and biomagnetic systems analyzed using cantilever magnetometry. *Nanotechnology*, 22(28):285715, 2011.

[7] J. Körner. *Gekoppelte Oszillatoren als neuartige Sensoren für Cantilever-Magnetometrie*. PhD thesis, Fakultät Elektrotechnik und Informationstechnik, Technische Universität Dresden, 2016.

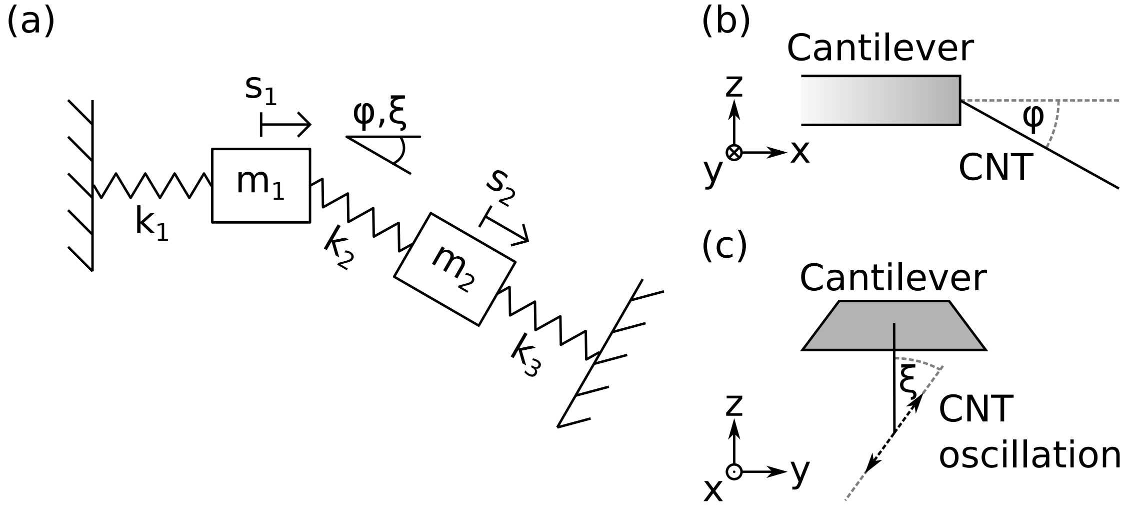

Figure 1: (a) Sketch of the angled coupled harmonic oscillator model employed to describe the non-aligned oscillation directions of the sensor's components. The angle between the two oscillation directions can be expressed by the angles ξ and φ. This results in a geometric reduction factor of $\epsilon = \cos(\varphi)\cos(\xi)$. (b) Sketch of the sensor illustrating the definition of angle φ as the structural inclination of the carbon nanotube (CNT) acting as nanocantilever with respect to the long axis of the cantilever. (c) Sketch of the sensor illustrating the definition of angle ξ by the non-aligned oscillation direction of the CNT. Please note that in (b) and (c) the given axes are valid for the mechanical considerations only and are different from the spatial frame of reference tied to the magnetic interactions in the main publication.
